# A Treatise on Dislocations and Fractures of the Joints

**Published:** 1823-03-01

**Authors:** 


*A Treatise on Dislocations and Fractures of the Joints.* By Sir Astley Cooper, Bart.

[Second and final Analytical Article. Concluded from p. 640, No. 11.]

Our readers are aware that, in our last number, we gave a very full analysis of that division of Sir Astley Cooper's work which embraced the important subject of dislocations and fractures at and about the hip-joint. The next series of accidents treated of by our author are—

*Dislocations of the Knee.* The articulating cavities of this joint are shallow, and on that account would be liable to dislocation, but their surfaces are broad, which counterbalances this liability. We shall not enter on the anatomy of this joint, which is ably sketched in the work before us, but proceed at once to the accidents to which it is obnoxious. The first is dislocation of the patella, which may take place in three directions—outwards, inwards, and upwards. Of the lateral dislocations, the outward one is the most frequent. "In either of these cases the ligament will be torn unless there be previous disease." The mode of reduction in both instances is as follows:—The patient is to be placed in a recumbent posture, while an assistant raises the leg by lifting it at the heel, thus relaxing the extensor muscles on the thigh. The surgeon is then to press down that edge of the patella which is most remote from the joint, be it one mode of luxation or the other, and this pressure raises the inner edge of the bone over the condyle of the os femoris, when it is immediately drawn into position by the action of the muscles. An evaporating lotion is first to be applied, and, in a few days, it may be bandaged.

*Dislocation upwards.* Here the ligamentum patellæ is torn through by the action of the rectus, and the effect of the injury is to draw the patella upwards upon the fore part of the thigh bone. The appearances are so obvious as to need no description. A smart inflammation follows this accident.

"In the treatment of this injury, local depletion and evaporating lotions are to be used from four to seven days from the accident, and then a roller is to be applied around the foot and upon the leg, to prevent its swelling; the leg is to be kept extended by a splint behind the knee, and a bandage composed of a leather strap is to be buckled around the lower part of the thigh; to this is to be attached another, which is to be carried on each side of the leg, and under the foot, and is to be buckled to the circular strap; thus the bone is gradually drawn down, so as to allow of an union of the ligament. In a month the knee may be slightly bent, and as much passive motion daily given as the patient is able to bear; by these means the ruptured ligament becomes united, and the patella retains its motion." P. 182.

While the bandage is worn the patient should persevere in the sitting posture, in order to keep the rectus muscle relaxed and prevented from acting on the patella.

*Dislocation of the Tibia at the Knee Joint.* This may happen in four ways—two of them incomplete and lateral—the others perfect luxations, the tibia being thrown either backwards or forwards. The lateral dislocations are but rare, and are easily reduced merely by extension.

*Dislocation forwards.* In this accident, which is uncommon, the external marks of the injury, when the patient is recumbent, are these—the tibia is elevated, the thigh bone is depressed and thrown somewhat to the side as well as backwards—the os femoris compresses the popliteal artery, and renders the pulsation in the anterior tibial imperceptible—the patella and tibia are drawn by the rectus muscle forwards. In a case of this kind, brought into Guy's Hospital in the year 1802, the limb was easily reduced by extending the thigh from above the knee, and by drawing the leg from the thigh and inclining the tibia a little downwards.

*Dislocation backwards.* This must be also a rare occurrence, as Sir Astley relates but one case, and that happened in the practice of Dr. Walshman. The appearances are—

a shortened state of the limb, a projection of the condyles of the os femoris, and depression of the ligament of the patella, the leg being bent forwards. It is to be reduced by extension.

*Partial Luxations.* Under extreme degrees of relaxation, or much synovial secretion, the ligaments of the knee-joint may become so much lengthened as to allow the cartilages to glide upon the surface of the tibia, particularly when pressure is made by the thigh bone on the edge of the cartilage. The most frequent cause of the accident is the toe striking against any projection, as the fold of a carpet, when the person is walking and the toe everted. He immediately feels pain in the knee, which he is unable completely to extend. The explanation we shall give in our author's own words.

"The semilunar cartilages which receive the condyles of the os femoris are united to the tibia by ligaments, and when these ligaments become extremely relaxed and elongated, the cartilages are easily pushed from their situation by the condyles of the os femoris, which are then brought into contact with the head of the tibia, and when the limb is attempted to be extended the edges of the semilunar cartilages prevent it. How then is the bone to be again brought upon the cartilages? Why, as Mr. Hey has advised, by bending the limb back as far as is possible, which enables the cartilage to slip into its natural situation, from the pressure of the thigh-bone being removed in the bent position, and the leg being brought forwards it can then be completely extended, because the condyles of the os femoris are again received on the semilunar cartilages." 191.

This plan is not invariably successful, as our author shews by an interesting case.

Of compound dislocations of the knee-joint Sir Astley has only met with one instance; he, therefore, concludes it to be one of very rare occurrence. No accident more imperiously demands immediate amputation. The case happened at Brentford, and Sir Astley amputated the limb on the same night. The patient recovered.

*Fractures of the Patella.* This bone is generally fractured transversely—rarely longitudinally. Compound fracture of this little bone but seldom occurs. In transverse cases the upper fragment is drawn, of course, from the lower by the action of the large muscles of the thigh, the degree of separation depending on the extent of laceration of the capsular ligament; for when the latter is but little torn, the separation will not be more than half an inch—which extends to four or five inches, where much injury is sustained by the ligament.

The accident may be at once known by the depression between the two portions of bone; by the fingers passing readily down to the condyles of the os femoris into the joint as far as the integuments will permit; and by the elevated portion of bone moving readily on the lower and fore part of the thigh. The power of extending the limb immediately after the accident is lost, and likewise that of supporting the weight of the body on that leg, if the person be standing, for the knee bends forwards from the loss of action in the extensor muscles. The pain of this accident is not very severe, and a simple fracture is not dangerous, for the constitution feels it but little. In a few hours after the accident, a considerable degree of extravasation of blood takes place upon the fore part of the joint, so that the appearance is livid from ecchymosis, but this is removed by absorption in a few days. Considerable inflammation and fever succeed, and there is a great degree of swelling in the fore part of the joint, both from the free secretion of synovia, and the effusion arising from inflammation. No crepitus is felt in this fracture, for the bones cannot be brought sufficiently near each other to give this general discriminating mark of other fractures." 202.

The accident arises from external violence or the sudden action of the muscles. The solution of the latter circumstance is not difficult. When the knee is bent, the patella is drawn down on the end of the condyles of the os femoris, so as to bring the upper edge of the bone forwards, and it is at that moment the patella is broken by the rectus muscle not acting in a line with the bone, but at right angles with it or nearly so, and upon its upper edge more particularly.

The union is generally by a ligamentous substance, whether the separation of the fractured portions be great or inconsiderable. The internal articular surface of the bone preserves its natural smoothness. Blood is immediately deposited in the place of the injured ligament, but is absorbed in a few days. Inflammation arises and pours out adhesive matter, which extends from one edge of the lacerated ligament to the other, and even between the bones, to each of which it is firmly united. Vessels shoot from the edges of the ligament, and organize the new substance. Our ingenious author has made many experiments on animals, in order to trace the mode of union in this bone. Although osseous union rarely takes place, the principle which is to guide the surgeon is to make the intervening ligament as short as possible.

"When called to this accident the surgeon places the patient in bed upon a mattrass, extends the limb upon a well padded splint placed behind the thigh and leg, to which it is tied, and which splint should be hollowed. The patient's body should be raised as much as he can bear to the sitting posture, to relax the rectus muscle. An evaporating lotion is to be then applied upon the knee consisting of Liq. Plumbi acetat. dilut. ℥v. with Spir. Vini. ℥i. and no bandage should be at first employed. The body should be slightly raised in bed to relax the rectus muscle, and the heel should be raised to bring up the lower portion of the patella. If, on the succeeding day or two, there be much tension or ecchymosis, leeches should be applied, and the lotion be continued; when, after a few days, the tension has subsided, then, and not till then, should bandages be employed." 208.

After detailing the means usually employed by others, Sir Astley states the following mode to which he gives the preference.

"A leather strap should be buckled around the thigh, above the broken and elevated portion of bone, and from this circular piece of leather, another strap is passed under the middle of the foot, the leg being extended, and the foot raised as much as possible. This strap is brought upon each side of the tibia and patella, and buckled to that which is fixed around the lower part of the thigh. The strap may be confined to the foot by a tape tied to it, and to the leg at any part in the same manner; and this is the most convenient bandage for the fractured patella, and for the patella dislocated upwards by the laceration of its ligament.—A roller is to be applied upon the leg." 209.

In this position, and thus confined, the limb is to be kept for five weeks in the adult, and for six weeks at a more advanced age. Then slight passive motion is to be begun with great circumspection, lest the ligament, if not firmly united, should give way and the bones again recede. After *passive* motion has been used for some time, the patient should be placed on a high seat and directed to swing the leg, so that the action of the rectus muscle may be gradually restored; for it is to be remarked that, in those who are kept in bed with the joint at rest, the power of bending and extending the limb is not acquired for many months. When the rectus muscle has been shortened, and the upper portion of bone is drawn from the lower, all disposition to action in the muscle ceases, and it does not recover its voluntary action until it becomes again elongated, which is effected after the union of the ligament, by bending the knee, from which point of elongation the muscle begins to contract.

It is obvious that the degree of approximation in the bones is of great importance. The less the distance the greater is the power which the muscle reacquires, and the less chance of falls and fractures afterwards.

*Perpendicular Fractures of the Patella.* A body was lately dissected in St. Thomas's Hospital, where both the patellæ were fractured longitudinally, and, although they were almost in contact, they were both united by ligament. These circumstances surprized our author, and he made several experiments on animals to elucidate the phenomenon. It resulted from these experiments that the want of osseous union in fractures, whether longitudinal or transverse, of the patella, arises from the parts not being in actual contact. Where they are kept in contact ossific agglutination takes place.

"In the longitudinal or perpendicular fracture of the patella, the best treatment consists in extending the leg, and in using local depletion, and evaporating lotions; in a few days a roller should be applied round the limb, and then a laced knee-cap, with a strap which buckles round the knee above and below the patella, with a pad on each side to bring its parts as nearly as possible into contact." 215.

*Compound Fractures of the Patella* are very dangerous, on account of the exposure and inflammation of the knee-joint. Two of the four cases related by Sir Astley died, the limbs being attempted to be saved. The two fatal cases were under the care of Mr. W. Cooper and Mr. Birch. The fourth case is a very interesting and important one.

Mr. Redhead, of Kennington Cross, aged 39, was thrown from his gig against a cart-wheel, by which the patella was shattered and the joint opened. Sir Astley was called in by Mr. Dixon, and found a wound on the fore part of the knee, through which he readily passed his finger into the joint, finding the patella shattered into several pieces, one of which was removed. The patient being of a spare habit, and placid inirritable disposition, it was agreed not to amputate. Knowing the difficulty of keeping the wound closed on account of the continued discharge of synovia, a suture was passed thro' the integuments, taking great care not to include any portion of the capsular ligament. Adhesive plaster and a roller finished the dressings, which were kept wet with spirituous lotions. The leg was placed in the extended position, and to be kept perfectly quiet. The patient to live on fruit. There was no swelling or inflammation of consequence, and the patient recovered without any untoward accident.

"If the laceration be extensive, or the contusion very considerable in these cases, the operation of amputation will be required; but if the wound be small, and the patient be not irritable and no sloughing of the integuments or ligament is likely to occur from the nature of the accident, it will be best to try to save the limb; and the treatment of Mr. Redhead's case is that which I should pursue. The principal object is to produce adhesion immediately, and every means in our power must be used to effect it. I know well that sutures are generally objectionable, and I never employ them, if I can possibly succeed without them, but in moveable parts, in those which are unsupported, and in those through which a secretion is liable to force its way, they are not only justifiable but highly necessary." 219.

*Oblique Fractures of the Condyles into the Joint* are of rare occurrence, and generally produce deformity. They are known by the great swelling of the joint, by the crepitus, and by the deformity with which they are attended. Whether the internal or external condyle be broken, the treatment will be the same. The limb is to be placed on a pillow, in the straight position, and evaporating lotions and leeches are to be used to subdue the swelling and inflammation. When this is effected, a roller is to be applied round the knee, and a piece of stiff pasteboard, about sixteen inches long, and wide enough to extend entirely under the joint, passing on each side of it, so as to reach the edge of the patella, is to be applied, having been previously dipped in warm water, and afterwards secured by a roller. In five weeks, passive motion may be gently employed to prevent anchylosis. Our author relates a fatal case of compound fracture of this kind, and he has known instances of the simple fracture proving mortal in aged persons.

*Oblique Fractures of the Thigh-Bone just above the Condyles* are formidable injuries in respect to the future form and use of the limb, being very liable to produce deformity, and prevent flexion of the knee-joint. We shall give the pathology in Sir Astley's own words.

"The appearances which the accident produces are, that the lower and broken extremity of the shaft of the bone projects, and forms a sharp point just above the patella, which pierces the rectus muscle, threatens to tear the skin, and sometimes does so; whilst the patella, tibia, and condyles of the os femoris sink into the ham, and are drawn upwards behind the broken extremity of the shaft of the os femoris." 226.

Several interesting cases are related in elucidation, but these we must pass over. To obviate the evils attendant on this unfortunate accident, Sir Astley has had an apparatus constructed to preserve the thigh in a constant state of extension, of which he has given a plate. The leg is to be first bent to draw the rectus muscle over the broken extremity of the bone, and then the apparatus is to be applied, and the limb is to be preserved in a constant state of extension in the straight position.

*Fracture of the Head of the Tibia.* If the fracture extend into the knee-joint, the treatment is the same as for oblique fracture of the condyles of the femur, that is—the straight position, a roller to press one part of the broken surface against another—and, thirdly, a splint of pasteboard to assist in the preservation of that pressure. Early passive motion will here also be proper to prevent anchylosis.

If the fracture of the tibia be oblique, but not into the joint, Sir Astley recommends the limb to be placed upon the double inclined plane, the weight of the leg keeping the limb constantly extended, and thus bringing the bones into as accurate apposition as the nature of the fracture will permit.

*Dislocations of the Caput Fibulæ.* This accident may arise from relaxation, or from external violence. Our author has seen but one instance of the latter, and that was connected with compound fracture of the tibia.

"Dislocations of the head of the fibula from relaxation, are more frequent than those which occur from violence, and the head of the bone is in these cases thrown backwards, and is easily brought into its natural connexion with the tibia, but it directly again slips from its position. This state produces a considerable degree of weakness and fatigue in walking, and the person suffers much from exercise. As in these cases there is a superabundant secretion of synovia, and a distension of ligament, repeated blistering is required to promote absorption; and afterwards a strap is to be buckled around the upper part of the leg, to bind the bone firmly in its natural situation, to which a cushion may be added behind the head of the bone, to give it support, and at least prevent the increase of the malady." 237.

We shall pass over the very important section in our excellent author's work, on Dislocations, simple and compound, of the Ancle-joint and Foot, as we have some idea of introducing the subject as a separate article in a subsequent number of the Journal. It has already formed a distinct analytical paper in our quarterly series. We shall, therefore, pass on to other subjects.

*Dislocations of the Clavicle.* These are easily enough understood and reduced. Thus, when the sternal extremity is dislocated forwards, the clavicle is easily returned to its place by pulling the shoulder backwards, by which the end of the bone falls into its place, whence, however, it will start forward again if pressure be not made on the fore part of the bone. The principle, therefore, upon which the extension is made, is to draw the scapula as far from the side as is practicable without inconvenience; and, secondly, to support the arm, to prevent the weight from influencing the position of the bone. The first of these objects is effected by the clavicle bandage, which is a modification of the common figure of 8 bandage—the second object is obtained by putting the arm in a sling.

Here our author relates a very daring operation performed by the late Mr. Davie, surgeon, of Bungay, in the removal of an inch of the sternal extremity of the scapula, in a case of distorted spine, where the said extremity of the bone was thrown by an accident behind the sternum, pressing so much on the œsophagus, as to produce extreme difficulty of deglutition. The dysphagia was removed, and the patient lived six years afterwards.

The dislocation of the scapular extremity of the clavicle is much more frequent of occurrence. The easiest mode of detecting this accident is to place the finger upon the spine of the scapula, and to trace this portion of bone forward to the acromion in which it ends. The finger is stopped by the projection of the clavicle over it, and so soon as the shoulders are drawn back, the point of the clavicle sinks into its place, re-appearing when the shoulders are let go. In this injury, the capsular ligament is necessarily torn through, as well as the external ligament going from the coracoid process to the clavicle. It is scarcely probable, Sir A. thinks, that the clavicle should be dislocated in any other direction than upwards—at least, he has never seen an instance of its gliding under the acromion. The cause of this accident is a fall upon the shoulders, by which the scapula is forced inwards towards the ribs.

"In the treatment of this accident, I adopt the following plan:—The assistant standing behind the patient, puts his knee between his shoulders, and draws them backwards and upwards, when the clavicle sinks into its socket. A thick cushion is then placed in each axilla, with three views; First, to keep the scapula from the side: Secondly, to raise the scapula: Thirdly, to defend the axillæ from being hurt by the bandages: on which last account, a cushion is employed on each side. Then the clavicle bandage is applied, and its straps should be sufficiently broad to press upon the clavicle, scapula, and the upper part of the os humeri, to keep the former down, the scapula upwards and backwards, which is the chief object, and the arm backwards and elevated. To secure these objects more effectually, the arm is to be suspended in a short sling, by which it is made to support the scapula in its proper situation." 407.

With the utmost care and attention, still some slight deformity will remain, which should be stated to the patient at the beginning, to prevent reflections afterwards.

*Dislocations of the Os Humeri.* Sir Astley prefaces this section with an anatomical description of the mechanism of this joint, into which it will not be necessary for us to enter. The head of the humerus is liable to be thrown from the glenoid cavity of the scapula in four directions—three of these dislocations being complete, and one being only partial. 1st, downwards and inwards into the axilla, the bone resting on the inner side of the inferior costa of the scapula:—2d, forwards under the pectoral muscle, the head of the bone being placed below the middle of the clavicle, and on the sternal side of the coracoid process:—3d, backwards, the head of the bone being felt and distinctly seen forming a protuberance on the back and outer part of the inferior costa of the scapula, the head of the humerus itself being situated upon its dorsum:—4th, partial, the anterior portion of the capsular ligament being torn through, the head of the bone resting against the coracoid process of the scapula on its outer side.

The dislocation downwards into the axilla is extremely common, and its characters are obvious enough. A hollow is producod below the acromion—the natural roundness of the shoulder is destroyed—the arm is elongated—the head of the bone can be felt in the axilla, "but only if the elbow be considerably removed from the side"—the motion of the arm is nearly lost, except forwards and backwards as it hangs by the side—some crepitus is occasionally felt on moving the limb, in consequence of inflammatory effusion and the escape of synovia—the central axis of the arm is changed, the central line running into the axilla—occasional numbness of the fingers. These are the essential characteristics, the principal of which are the fall of the shoulder, the presence of the head of the bone in the axilla, and the loss of motion in the joint. But a few hours make these appearances much less decisive, from extravasation and swelling. These last subsiding the marks of the injury again become decisive. It is at this period, generally, that metropolitan surgeons are consulted, and when dislocation is then detected, it is their duty to state candidly to the patients that the difficulty of detection is exceedingly diminished by the subsidence of tumefaction and inflammation. Here Sir Astley gives three interesting dissections of dislocations downwards—one immediately after the injury, another at a distance of five weeks from the date of the accident. These we must pass over.

Various are the means of reduction which have been employed by surgeons; and they must vary according to the circumstances of the case. That which our experienced author usually adopts in private practice in all recent cases, is by the heel in the axilla.

"The patient should be placed in the recumbent posture, upon a table or a sofa, near to the edge of which he is brought; the surgeon then binds a wetted roller round the arm immediately above the elbow, upon which he ties a handkerchief; then, with one foot resting upon the floor, he separates the patient's elbow from his side, and places the heel of his other foot in the axilla, receiving the head of the os humeri upon it, whilst he is himself in the half sitting posture by the patient's side: He then draws the arm by means of the handkerchief, steadily for three or four minutes, when, under common circumstances, the head of the bone is thus easily replaced; but if more force be required, a long towel may be applied instead of the handkerchief, by which several persons may pull, the heel still remaining in the axilla. I generally bend the fore arm nearly at right angles with the os humeri, because it relaxes the biceps, and consequently diminishes its resistance. I have, in many cases, extended from the wrist, by tying the handkerchief just above the hand, but more force is required than in the former mode, although it has this advantage, that the bandage is less liable to slip. In recent cases, it very rarely happens that this mode of extension fails, and it is so easily applied in every situation, that I have recommended all our young men to employ it in the first instance, when called to this accident." 427.

Where, however, the muscles are very powerful, and the accident has occurred several days, allowing the muscles to become permanently contracted, the reduction requires a greater degree of force than can be applied in the above manner. The patient must be placed in a chair, and the scapula fixed by means of a bandage which allows the arm to pass through it, (the one used at Guy's is a girt buckled on the top of the acromion, so as to raise the bandage high in the axilla, thus more completely fixing the scapula, the principal object to be attended to, else all efforts will be inefficient,) a wetted roller is next to be bound around the upper arm, just above the elbow, and upon this a very strong worsted tape is to be fastened, (in a manner hereafter to be described when speaking of the reduction of dislocated fingers,) the arm should then be raised at right angles with the body; or a little higher, if there be much difficulty of reduction; two persons should then draw from the bandage affixed to the arm, and two from the scapula bandage, with a steady, equal, and combined force. Jerking should be entirely avoided, and every aim at quick reduction discountenanced. "*Slowly and steadily* should be the word of command from the surgeon," who after the extension has been made some minutes, should place his knee in the axilla, resting his foot upon the chair; he then raises his knee by extending his foot; and placing his right hand upon the acromion, pushes it downwards and inwards, when the head of the bone usually slips into its natural position. A gentle rotatory motion, during the extension, is sometimes productive of diminished opposition in the muscles. These means failing we must have recourse to a third mode of reduction, viz. the *pullies*—not with the view of employing *greater* force, for that could be obtained by more persons; but with the view of applying *gradual and equal* force, without jerks and unequal extension, which are sure to occur when manual strength is employed for any length of time.

"For the application of the pulley, the patient sits between two staples, which are screwed into the wainscot, on each side of him; the bandages are then applied, precisely as in the former mode, as when the extension is made by men, and the force is applied in the same direction; the surgeon should first draw the pulley, as the class of people whom he usually has to assist him, being ignorant of the principle upon which it is employed, would use too great violence, he should draw gently and steadily, until the patient begins to complain of pain, and then cease keeping up that degree of extension, and conversing with the patient to direct his mind to other objects. In two or three minutes, more force should be applied, and continued until pain be again complained of, when the surgeon should again cease to increase the force; and thus he should proceed for a quarter of an hour, at intervals slightly rotating the limb. He should, when he has applied all the extension he thinks right, give the string of the pulley to an assistant, desiring that degree of extension to be supported; then putting his knee in the axilla, and resting his foot upon the chair, he gently raises and pushes back the head of the bone towards the glenoid cavity, when the bone passes into its socket; but generally without the snap which is heard when other means are employed, yet both the surgeon and the patient are aware of some motion of the head of the bone at the time." 431.

In hospital practice Sir Astley orders the patient to be bled, and put into a warm bath at the temperature of 100 or 110, giving him a grain of tartrite of antimony every ten minutes until he becomes faint. He is then wrapped in a blanket, placed in a chair, and the extension made before the muscles have time to recover. When the reduction has been effected, a small cushion should be placed in the axilla and fixed there by a stellate bandage, to prevent the head of the bone again slipping from its situation. The arm is also to be supported in a sling.

There is a fourth mode of reduction, *by the knee in the axilla*, which is applicable to recent dislocations, to delicate females, and to very old, relaxed, and emaciated persons.

"The patient is seated upon a low chair, the surgeon then placing himself by him, separates the dislocated arm from the side sufficiently to admit his knee into the axilla, and resting his foot upon the side of the chair, he places one hand upon the os humeri, just above the condyles, and the other upon the acromion scapulæ, he then pulls down the arm, over the knee, and in this manner reduces the dislocation." 433.

The ambe cannot be recommended where the extension is to be of any continuance, as its fixed point of action is on the ribs of the patient, and cannot long be borne without injury.

Mr. Kirby's process of reduction, of which we have given a plate in the January (1820) number of this Journal is mentioned by Sir Astley as an ingenious method.

*Dislocation forward.* The phenomena of this species are more distinctly marked than in the former. The acromion is more pointed, and the hollow below it more considerable. The head of the os humeri can be distinctly felt, and in thin persons seen. The coracoid process of the scapula is placed on the outer side of the head of the bone—the arm is somewhat shortened—and it is thrown more from the side and farther back than in dislocation downwards—the axis of the limb is much altered, being thrown inwards towards the middle of the clavicle. "But the strongest diagnostic marks of this dislocation are, the head of the bone being below the clavicle—and the elbow separated from the side and thrown backwards."

*Reduction.* The foot in the axilla, as in the former case, will generally succeed in this; but here the foot should be placed more forward to press on the head of the bone, and the arm should be drawn obliquely downwards and a little backwards. Where the accident has happened some days, continued extension will generally be necessary, and then the pullies must be employed.

"The same bandage is required as in the dislocation in the axilla, whether the power used, be through the medium of pullies or of men. The arm should be bent to relax the biceps muscle; but the principal circumstance to be considered is, the direction in which the bone is to be drawn, and the best is slightly downwards, for if it be drawn in the horizontal direction, the head of the os humeri is pulled directly against the coracoid process of the scapula, and a difficulty created which may be avoided." 439.

*Dislocation on the Dorsum Scapulæ.* Here the head of the humerus is thrown upon the posterior surface of the inferior costa of the scapula. It cannot be mistaken, as there is a protuberance formed by the bone upon the scapula which immediately strikes the eye. Only two cases have occurred in Guy's Hospital during 38 years. A case is stated from the practice of Mr. Toulman of Hackney, and two from the practice of Mr. Coley of Bridgenorth, an ingenious and very able practitioner. The reduction is not difficult, and does not materially differ from the process already described.

*Partial Dislocation,* Sir Astley thinks, is not of very rare occurrence, and is distinguished by the following marks:—

"The head of the bone is drawn forwards against the coracoid process; there is a depression opposite the back of the shoulder-joint, and the posterior half of the glenoid cavity is perceptible from the advance of the head of the bone; the axis of the arm is thrown inwards and forwards; the under motions of the limb are still capable of being performed; but its elevation is prevented, by the head of the humerus striking against the coracoid process; there is an evident protuberance formed by the head of the bone in its new situation, which is felt readily to roll when the arm is rotated." 446.

The mode of reduction is the same as that for the dislocation forwards; but it is necessary to draw the shoulders backwards to bring the head of the bone to the glenoid cavity. When the dislocation is reduced the shoulder should be bound back by a clavicle bandage, else the bone will immediately slip forward against the coracoid process. An injury of excessive violence will sometimes occasion the head of the bone to be forced through the integuments in the dislocation forwards, an instance of which is inserted from the practice of Mr. Saumarez and Mr. Dixon of Newington. Such cases require an immediate reduction by the means already pointed out for the forward dislocation, and then the wound dressed secundum artem.

There are some fractures about the shoulder-joint that are liable to be confounded with dislocations.

*Fracture of the Acromion.* The roundness of the injured shoulder is lost—the head of the os humeri sinks towards the axilla—a depression at the junction of the spine of the scapula with the clavicle is felt, from the fall of the fractured portion. If the surgeon raises the arm from the elbow, so as to put the deltoid muscle in motion, the natural form of the shoulder is directly restored; but the deformity returns immediately the arm is again suffered to fall.

"Fracture of the acromion scapulæ will unite by bone, but it generally unites by ligamentous substance, in consequence of the difficulty which exists in producing adaptation, and in preserving the limb perfectly at rest during the period required for union. In the treatment of this accident, the head of the os humeri is the splint which is employed to keep the acromion in its natural situation; and with this view, the elbow is raised, and the arm is fixed; thus the bone will be raised to the under surface of the acromion, and if it be kept steadily in that position, it will support and keep in its place, the broken process. The deltoid muscle should be also relaxed, and this is best effected by a cushion being placed between the elbow and the side, for if the elbow be brought close to the side, the broken acromion is further separated. The arm should be raised as mnch as is possible, and the elbow be carried a little backwards, and then bound to the chest by a roller; in this position it should be kept firmly fixed for three weeks, doing every thing to prevent any motion of the bone. Very little inflammation succeeds this accident, and the disposition to ossific union is very feeble in the separated portions of bone.

"In this case, if a pad be placed in the axilla, the broken portion becomes widely separated from the spine of the scapula, by its having the effect of throwing out the head of the os humeri." 456.

But the accident most liable to be mistaken for dislocation is fracture through the narrow part of the cervix scapulæ, immediately opposite the notch of the superior costa; by which the glenoid cavity becomes detached from the scapula, and the head of the bone falls with it into the axilla. The shoulder in this case falls—there is a hollow below the acromion, from the deltoid muscle sinking—and the head of the os humeri can be felt in the axilla. The degree of deformity in this accident depends on the extent of laceration of the ligament which passes from the under part of the spine of the scapula to the glenoid cavity, and which is not generally described in anatomical books. If this be torn, the glenoid cavity and head of the os humeri fall deeply into the axilla, but the displacement is much less if this remain whole.

"The diagnostic marks of this accident, are three; *first*, the facility with which the parts are replaced; *secondly*, the immediate fall of the head of the bone into the axilla, as the extension is removed; and *thirdly*, the crepitus which is felt at the extremity of the coracoid process of the scapula, when the arm is rotated. The best method of discovering the crepitus is, for the surgeon's hand to be placed over the top of the shoulder, and the point of the fore finger to be rested on the coracoid process, the arm being then rotated, the crepitus is directly perceived, because the coracoid process being attached to the glenoid cavity, and being broken off with it, although itself uninjured, the crepitus is communicated through the medium of this process." 458.

The treatment consists, *first*, in carrying the head of the os humeri outwards—and *secondly*, in raising the glenoid cavity and arm. The *former* is effected by a thick cushion being placed in the axilla, which presses the head of the bone and glenoid cavity outwards, and this may be confined by the clavicle bandage—the *latter* is produced by placing the arm in a short sling, and then the raised head of the os humeri supports the glenoid cavity and cervix scapulæ, keeping it steadily in its place till union is effected, which generally requires ten or twelve weeks in the adult.

*Fracture of the Neck of the Humerus.* This bone is sometimes broken just below its tubercles through the cervix. It is more frequent in the young and in the old—rarely occurs in middle age. In this accident the head of the bone remains in its place; but the body of the humerus sinks into the axilla, where its extremity can be felt, and it draws down the deltoid muscle, so as to lessen the roundness of the shoulder.

"The best diagnostic marks are the following: embrace the head of the os humeri with the fingers and fix it, then rotate the arm at the elbow, and it will be found, that the head of the bone does not obey the rotatory motion, as it is separated from the body of the humerus by the fracture, which is in this case, external to the capsular ligament. The bone, in these cases, unites in from three to six weeks, according to the age of the patient." 460.

*Treatment.* Apply a roller from the elbow to the shoulder joint—place a splint on the inner and outer side of the arm—confine all by means of a roller—place a cushion in the axilla, and support the arm *gently* in a sling.

*Dislocations of the Elbow Joint.* This section is prefaced by a good anatomical sketch of the mechanism of the part, for which we must refer to the original. The species of dislocations are five:—1st, Both bones dislocated backwards. 2. Both bones laterally. 3. The ulna dislocated from the radius. 4. The radius dislocated forwards. 5. The radius dislocated backwards.

1. *Both Bones dislocated backwards.* The shape of the elbow is altered—considerable projection posteriorly formed by the ulna and radius above the natural situation of the olecranon—hard swelling at the fore part of the joint behind the tendon of the biceps—hand and forearm are supine, and cannot be rendered entirely prone—flexion of the joint in a great degree lost.

*Reduction.* "The patisnt is made to sit down upon a chair, and the surgeon places his knee on the inner side of the elbow-joint, in the bend of the arm, and taking hold of the patient's wrist, he bends the arm; at the same time, he presses on the radius and ulna with his knee, so as to separate them from the os humeri, and thus the coronoid process is thrown from the posterior fossa of the humerus; and whilst this pressure is supported by the knee, the arm is to be forcibly but slowly bent, and the reduction is soon effected. It may be also accomplished by placing the arm around the post of a bed, and by forcibly bending it whilst it is thus confined. I have also reduced the limb by making the patient, whilst placed upon an elbow chair, put his arm through the opening in its back, and then having bent the arm, the body and limb being thus well fixed, the reduction of the dislocation was easily effected." 469.

The arm should now be bandaged in the bent position—

evaporating lotions applied—and the limb supported in a sling, the forearm bent at rather less than a right angle with the upper arm. A splint may be placed in a sling for the better support of the limb.

2. *Lateral Dislocation.* The projection of the ulna backwards is greater than in the former dislocation—the radius forms a protuberance behind and on the outer side of the os humeri, so as to produce a hollow above it—the rotation of the head of the radius is distinctly felt by rolling the hand. The reduction may be easily effected, as in the former dislocation, by bending the arm over the knee, even without particularly attending to the direction of it inwards or outwards.

3. *Dislocation of Ulna backwards.* The limb is much deformed by the twisting inwards of the forearm and hand—the olecranon projects and can be felt behind the os humeri—extension of the arm is impracticable—it cannot be bent to more than a right angle. These are its characteristics.

"This dislocation is more easily reduced, than that of both bones; and the best method is, by bending the arm over the knee, and to draw the fore arm downwards; it may then be easily reduced, as not only the brachialis muscle will act in resistence, but the radius, resting against the external condyle, will push the os humeri backwards upon the ulna, when the arm is bent." 474.

4. *Dislocation of Radius forwards.* Our author has seen six examples of this accident. *Symptoms.* Forearm slightly bent, but cannot be brought to a right angle with the upper; nor can it be completely extended. When it is suddenly bent the head of the radius strikes against the fore part of the os humeri, and produces so sudden a stop to its motion as at once to convince the surgeon it is one bone striking against the other. Neither pronation nor supination can be completely performed. If the thumb be carried into the fore and upper part of the elbow joint, the head of the radius may be there felt, and if rotation of the hand be attempted, the bone will be perceived to roll. "This last circumstance, and the sudden stop to bending the arm, are the best diagnostic marks of the injury." Of the six cases related, it appears that only two were reduced. The reduction, in fact, is exceedingly difficult, and has foiled a Cline and a Cooper. In two cases, however, Sir Astley was completely successful. Our author thinks the extension should be made from the hand alone (a suggestion which he candidly attributes to one of his pupils, Mr. Williams) "as from the connexion of the hand with the radius, that bone alone is acted upon, and by excluding the ulna from the force applied, the radius sustains the whole extension." It is also right in making the extension that the hand be rendered supine, as this position draws the head of the radius from the upper part of the coronoid process of the ulna, upon which it is otherwise directed, and then to draw the fore arm by pulling the hand, and by fixing the os humeri.

The dislocation of the radius *backwards* our author has never seen in the living subject. One instance of the kind was brought into the dissecting room in the year 1821, from which a drawing is given by Sir Astley.

*Fractures of the Elbow Joint.* The condyles of the os humeri are sometimes obliquely broken off just above the joint, and the appearance produced is so similar to the dislocation of the radius and ulna backwards, that the two accidents are very liable to be confounded together. The mode of distinguishing the two injuries is, by all the marks of dislocation being removed by extension, and by their return so soon as the extension is withheld. In these accidents too, a crepitus can generally be felt when rolling the fore-arm upon the humerus. It is of much more frequent occurrence in children than in grown persons, though it happens at all periods of life.

" Its treatment consists in bending the arm, and drawing it forwards to effect replacement, and then a roller should be applied whilst it is in the bent position. The best splint for it is one formed at right angles, the upper portion of which, is to be placed behind the upper arm, and the lower portion under the fore arm; a splint must also be placed upon the fore part of the upper arm, and straps to confine both; evaporating lotions should be used; and the arm kept in a bent position by a sling. In a fortnight, if the patient be young, passive motion may be gently begun, to prevent the occurrence of anchylosis; and in the adult, at the end of three weeks, a similar treatment is to be pursued." 482.

There is sometimes considerable loss of motion, notwithstanding the most judicious means have been employed.

*Fracture of the internal Condyle of the Humerus.* This portion of bone is frequently broken off, in which case the ulna projects backwards, having lost its support. If the fore-arm be extended, the hand becomes twisted inwards towards the side; but, upon flexion, these appearances are removed. The above circumstances, and a crepitus on bending or extending the arm, will distinguish this accident from others. The treatment is the same as that for oblique fractures above the condyles of the humerus.

*Fracture of the Olecranon.* This process is not unfrequently broken off, and the symptoms are so evident that they can scarcely be mistaken. There is pain, and a soft swelling at the back of the elbow, through which the surgeon's finger readily sinks into the joint—the olecranon can be felt in a detached piece—elevated, and moveable from side to side, but with great difficulty drawn downwards—bending of the arm renders the separation greater—the power of extension is nearly lost—there is a proneness to semi-flexion—swelling and ecchymosis continue for some days after the accident—rotation of the radius upon the ulna is still preserved—no crepitus.

When the fracture takes place, the triceps muscle draws up the detached piece of bone from half an inch to two inches from the ulna, the extent of the separation depending on the degree of laceration in the capsular ligament, and the ligamentous band stretching from the side of the coronoid process to the olecranon. Sir Astley has made some interesting experiments on animals, in order to elucidate the nature of this injury, and the means of reparation, for which we must refer to the volume itself.*

"The treatment of this accident is as follows, but it is to be regulated by the degree of injury. If there be much swelling and contusion, it is right to apply for two or three days evaporating lotions and leeches; and after the inflammation is reduced, a bandage should be applied; but in those cases where but little violence is done to the limb, it should be at once secured by bandage. The principle of the treatment is, to preserve the power of the limb, by making the separation of the bones as slight as possible, and consequently to shorten their ligamentous union; and secondly, to restore the natural motions of joint. If the swelling and inflammation do not prevent it, the surgeon is to place the arm in a straight position, and to press down the upper portion of the fractured olecranon, until he brings it in contact with the ulna; a piece of linen is then laid longitudinally on each side of the joint, a wetted roller is applied above the elbow, and another below it, the extremities of the linen are then to be doubled down over the rollers, and tightly tied, so as to approximate them, thus the bones are brought and held together; a splint well

---

* We may, however, introduce a short extract shewing the *results* of Sir Astley's experiments.

"Therefore, this bone, like the extremity of the os calcis when it is broken off, is detached by the action of muscles, and ligamentous union takes place from want of adaptation; but a different cause exists, where bony union is deficient in fractured bones within joints, in the neck of the thigh-bone; in the coronoid process of the ulna; and in the extremity of the external condyle of the os humeri; in which injuries, the want of union depends upon the diminished support the fractured parts receive, and that little being through the medium of blood vessels intended for the nourishment of ligament." 488.

padded is to be applied upon the fore part of the arm, to preserve it in a straight position, and is to be confined to it by a circular bandage; the whole is to be frequently wetted with spirits of wine and water." 490.

In a month the splint is to be removed and passive motion begun, with great caution at first. In *compound fractures* of this bone, the edges of the skin must be brought into exact apposition—lint imbued in blood is to be applied on the wound with adhesive plaster—and union by adhesion effected if possible. In other respects, the treatment is the same as in simple fracture.

*Fracture of the external Condyle of the Humerus* is not uncommon among children; but rarely occurs in the adult, or in old age. It is recognized by swelling on the external condyle, and pain on pressure, as well as on extension or flexion of the elbow joint; "but the principal diagnostic sign is the crepitus produced by the rotatory motion of the hand and radius." In two preparations at St. Thomas's Hospital the union is by ligament.

"It is obvious therefore," says our author, "that this principle of ligamentous union, extends to all detached portions of bone within a capsular ligament; its vitality being supported merely by the ligament within the joint." 492.

*Treatment.* A roller is to be applied around the elbow, and above and below the joint—an angular splint is then to be applied, which should admit the elbow, and extend behind the upper arm, and receive the fore arm so as to support it—a roller should next be bound over the whole to keep it firmly fixed. The splint should be worn for three weeks, when passive motion is to be begun, with great gentleness at first, and to be very gradually increased.

*Compound Fracture and Dislocation of the Elbow Joint.* These generally happen through the internal condyles of the humerus, the fracture taking place in an oblique direction into the joint. If judiciously treated, the constitution will generally be able to support even the most severe accidents of this kind, some very interesting cases of which are detailed by our author, for which we must refer to the original.

"In all cases of this accident, the arm should be kept in the bent position, for as anchylosis in a greater or lesser degree, is sure to be the consequence, it is attended with much less inconvenience in this position, than in any other. If the bones be much comminuted, and the wound large, all the detached portions of bone should be removed; but in old people, when much injury is done, there is often not sufficient strength to support the adhesive process, and amputation should be recommended. The edges of the wound should be kept together by placing a piece of lint dipped in blood over them, supported by adhesive plaster, and a bandage lightly applied, wetted with spirits of wine and water." 498.

We perceive, from our limits, that we must leave some portions of the work unnoticed, and we shall, therefore, pass over the sections on dislocations and fractures of the wrist, carpal and metacarpal bones, fingers, &c. in order to concentrate our attention more on certain injuries about the trunk of the body.

On dislocation of the ribs, our author says but little, apparently from a conviction that such an accident is extremely rare. A person may, he thinks, by falling on his back upon some pointed body, receive such a blow on the ribs, as may drive them from their articulations. This injury would produce the same symptoms as fracture of the costæ:—their motions would be painful, and respiration difficult. The treatment would be the same as for fractured ribs, viz. blood-letting, and the application of a circular bandage.

### Injuries of the Spine.

Dislocations of the spinal column are represented by some authors as of frequent occurrence—but Sir Astley suspects that they are extremely rare, as he has never seen an instance of the kind unaccompanied by fracture of the bodies or processes of the vertebræ. He does not, however, deny the possibility of dislocation, especially of the cervical vertebræ, the articulating processes of which are placed more obliquely than those of the other vertebræ.*

---

* We shall here introduce some cases in illustration of this point, which were lately transmitted to us by Mr. Fielding, of Hull.

" *On Partial Luxation of the Vertebræ.*

" Though I presume it is almost certain, that *complete luxation* of the vertebræ cannot take place independent of fracture, yet, from some cases which have fallen under my own observation, it seems to me to be equally certain, that *partial luxation* may take place without it. I have seen four cases in which, from falls, the *ligamentum nuchæ* was injured, and the last vertebra of the neck was thrown partially forward upon the uppermost dorsal. These cases came too late under my notice to be remedied —but the effect of the injury was quite apparent;—the spinous process of the *first vertebra dorsi* was much too prominent—the head, permanently bent down towards the sternum, could not be raised to the erect position. The two following cases illustrate more clearly the nature of the sort of accident I mean.

" J. M——, æt. 14, a delicate youth, gave me the following account: Yesterday evening, walking at a quick pace home, he set his foot upon some soft substance on the flags and slipped; apprehensive of falling, he made a sudden effort to recover himself, and succeeded; but, on the in-

The effects resulting from violence done to the spinal chord are very similar to those which are produced by injuries to the brain, viz. concussion, extravasation, fracture, fracture with depression, suppuration, and ulceration.

*Concussion.* The effect of this, if in a severe degree, is generally paralysis of the parts beneath; the person often recovering, in a gradual manner, the motion and sensation of the parts. A case is related, where cupping, purging, and instituting a drain from the back, restored a severe injury of this kind.

*Extravasation.* A very severe blow upon the vertebræ will sometimes produce extravasation upon the spinal chord, but more frequently upon the sheath in which it is contained. Of late years, since it has been the custom, in examining dead bodies, to saw off the spinous processes of the vertebræ, in cases of severe injury, blood has been, several times, found on the outer side of the spinal sheath, and, in one instance, upon the spinal marrow, just above the cauda equina. We shall give the following case entire.

---

stant, felt violent pain in the neck, and could not bring his head into the erect position. On arriving at home, he was rubbed for supposed sprain and went to bed He passed a sleepless night, being obliged to lie always upon his back. Next morning he still could not raise his head, and on attempting to pursue his avocation in the office, found his head become dizzy and that his vision was indistinct. On his return home this morning, Sept. 10, 1822, I saw him, and found that his head was immoveably inclined to the left side, so as to be almost in contact with the shoulder, which was elevated to meet it—complained of head-ache and dizziness. On examining the spine, I at once discovered, that the spinous process of the last vertebra of the neck was distorted towards the right side, I felt (or supposed I felt) that the inferior oblique process was, also, too prominent on the same side. Without giving any notice whatever to the patient, I placed the point of the thumb of my left hand, as accurately as I could, firmly upon the oblique process, and seizing the head with the other hand, drew it quickly, but not violently, towards the right shoulder, pressing firmly upon the vertebra with my thumb at the same time; the bone was immediately replaced, the young man jumped up suddenly from his chair, and cried out "sir I am well," and immediately, at my desire, moved his head and neck in every direction with little inconvenience. He was perfectly well and returned to his employment the next day."

"*Case* 2. A poor girl, æt. 14, was brought to my house, Sept. 16, 1822—her mother said that she was thrown down in the street yesterday evening, by some person running suddenly against her—she has never since been able to raise the head to the erect position. With the exception that the head inclines to the right side—the appearances are nearly the same as described in the former case—a similar method of reduction was employed, and the patient was cured at once."—*Private Correspondence*.

"Master ——, a fine youth aged twelve years, in June 1814, was swinging in a heavy wooden swing, and just commencing the moving forward, was caught by a line which had got under his chin, by which accident his head was violently strained, and the whole of the cervical vertebræ; as however, the line slipt immediately off, he thought no more of it. Subsequently to the accident for some months, he was not aware of any pain or inconvenience, but his school-fellows observed he was less active than usual; instead of filling up his time by play, he would be laying on the school forms, or leaning on a style or gate, when in the fields. They were always teasing him on this account, and at last, he was persuaded himself, that he must be weaker than he used to be. From this time he continued to decline both in strength and power. About the middle of May following, he came to London. His complaints were occasional pains in the head, which were more severe and frequent about the back of his neck, (where a blister had been applied without relief,) and down his back. The muscles at the back of the head and neck were stiff, indurated, and very tender to external pressure. He felt pain in moving his head or neck in any direction; added to these symptoms, there was a great deficiency in the voluntary powers of motion, especially in the limbs.

"May 18th. Two setons were made in the neck, and he was ordered various medicines, without any being useful.

"May 29th. His complaints and the paralytic affection of his limbs, were getting much worse, added to which, he felt a most vehement hot burning pain in the small of his back. This, by the next day, was succeeded by a sense of extreme coldness in the same part. Some time after, the same pain occurred higher up in the back, and then disappeared. Pulse, and heat, natural.

"June 3rd. A consultation of Dr. Baillie, Dr. Pemberton, Mr. A. Cooper, and Mr. Heaviside, was held, and the application of mercury was determined on. The pil. hydr. was taken for a few days, but as it run off by the bowels, mercurial frictions were consequently preferred. He felt his limbs getting every day weaker, but his neck was more free from pain when moved, and he was more capable of moving it by his own natural efforts.

"June 7th. His respiration became laborious, he passed a bad night, on the following day all his symptoms increased, and at five in the afternoon, he expired.

"*Examination.* The whole contents of the head were carefully examined, and found perfectly healthy; but upon sawing out the posterior parts of the cervical vertebræ, the theca vertebralis was found overflowed with blood, which was effused between the theca and the enclosing canals of bone. The dissection being further prosecuted, this effusion extended from the first vertebra of the neck, to the second vertebra of the back, both included.

"The preparation only shews a small proportion of the effused blood which had become coagulated on the theca, as much of it escaped in the act of removal, it being fluid." 548.

The particulars of the above were obtained from Mr. Heaviside, whose splendid museum is ever found open for the good of the profession.

*Fracture of the Spine.* These accidents, even when the bones retain their situation, produce very extraordinary symptoms, by admitting unnatural variations in the positions of the spinal column. When there is fracture with displacement, the symptoms differ according to the seat of the fracture. These injuries our author divides into two classes—1st, those which occur above the third cervical vertebra—and 2dly, where the injury is below that bone. In the *first* class, the accident is almost always fatal, if the displacement be to the usual extent. Death, in the second class, occurs at various periods after the injury. The circumstance of the phrenic nerve taking its origin from the 3d and 4th cervical pair, is the cause of this difference. If the lumbar vertebræ be displaced, the lower extremities are rendered completely insensible—the power of volition is completely destroyed—the sphincter ani loses its function, and the fæces are passed involuntarily. The bladder is unable to contract, and the urine is retained till drawn off by the catheter. Nevertheless, the circulation goes on, though somewhat more languidly, and inflammation can be raised by blisters. It is curious, that priapism is a common phenomenon under such circumstances. In general, in fractures of the lumbar vertebræ, patients die in a month or six weeks after the injury. In fractures of the dorsal vertebræ, the symptoms are nearly similar; but the paralysis extends higher, and the abdomen becomes greatly inflated, from the diminution of nervous energy. Death sooner takes place here, than in lumbar fracture—the patient usually sinking in a fortnight or three weeks. "The period of existence is short or protracted, as the injury is near or distant from the cervical vertebræ, and as the displacement is slight or considerable, as well as the degree of injury the spinal marrow has sustained."

"Fractures at the cervical vertebræ below the origin of the phrenic nerve, produce paralysis of the arms, as well as of the lower parts of the body; but this paralysis is seldom complete; if it occurs at the sixth or seventh vertebra, the patient has some feeling and powers of motion, but if at the fifth, little or none; sometimes one arm is much more affected than the other when the fracture is oblique, and the axillary plexus of nerves is consequently, partially influenced. Respiration is in these cases, difficult, and performed wholly by the diaphragm, the power of the intercostal muscles being destroyed by the accident. The abdomen is also tumid from flatulency, as when the dorsal vertebræ have sustained injury. The other symptoms are the same as in fractures of the vertebræ below the cervical, as regards the lower extremities, the bladder, and the sphincter ani. Death ensues in these cases in from three to seven days, as it is seated in the fifth, sixth, or seventh vertebra. I have scarcely known the subject of this injury to live beyond a week, and but rarely to die on the second day, although they sometimes do if the fifth cervical vertebra has sustained the injury. I have already stated, that in fractures and displacements above the fourth cervical vertebra, death instantaneously follows." 556.

In the dissection of these cases, the spinous process of the displaced vertebra is depressed—the articular processes are fractured—the body of the vertebra is broken through—but, it rarely happens, that the displacement and separation occur at the intervertebral substance. The body of the vertebra is usually advanced from half an inch to an inch—between the vertebræ and the sheath of the spinal marrow, blood is extravasated; and, frequently, there is extravasation of blood on the spinal chord itself. The spinal marrow is compressed and bruised in slight displacements, and is torn through when the injury has been very extensive, the dura matral covering remaining entire. A *bulb* is formed at each end of the lacerated spinal marrow, which laceration is usually produced by the bony arch of the spinous processes. A very interesting case is related, from the practice of Mr. Harrold of Cheshunt, where the spine was broken at the lower part of the dorsal, or beginning of the lumbar, vertebræ. The principle on which Mr. Harrold proceeded, was to produce union of the bones by preserving the spine in perfect rest; to effect which, the patient was put into a fracture bed, where he had the means of evacuating the bowels without disturbance. The urine was drawn off daily by the catheter, for several weeks, after which, he was able to retain and discharge it when he pleased. At the end of six months, the following was the report of his health. His back straight, flexible, and apparently as strong as ever—retains and discharges his urine—has a stool once in three or four days—health and spirits good—but no sensation or voluntary motion in the lower extremities. He died in twelve months from the accident, owing to a sore on the tuberosity of the ischium, and disease of the bone. Sir Astley examined the body with great care, and the preparation is preserved in the museum of the College. The bodies of the first and second lumbar vertebræ had been fractured—the first having advanced, and the second having been forced backwards. The fracture had united by ossific matter, which had spread over the fore part of both vertebræ to a considerable extent, and a little had been deposited upon the dorsal vertebræ. The spinal canal had been much diminished by a portion of bone being forced into it from the first vertebra of the loins. This portion of bone had split the theca vertebralis into two, and divided the spinal marrow almost entirely—a bulbous projection of the latter appearing above and below the bone, formed by its divided extremities, which were separated nearly an inch from each other.

In respect to the treatmeut of these unfortunate cases, our author has little to offer that is at all consolatory. Rest will be essential to ossific union; but ossific union will not save the patient, if the spinal marrow be not relieved from pressure. The late Mr. Henry Cline took a scientific view of this subject. He considered the accident as similar to fracture with depression of the cranium—and to require the removal of that pressure. He, therefore, thought himself justified in making an incision upon the depressed bone, as the patient was lying upon his breast—he then raised the muscles covering the spinal arch, and applied a small trephine to the arch, cutting through it so as to remove the spinous process, and the arch pressing on the spinal marrow. The case did not succeed; and, unfortunately, he did not live long enough to prosecute the practice. The operation has lately been repeated by Mr. Tyrrel, at St. Thomas's Hospital, and, also, without success. We are convinced that the fatal issue, in both cases, was owing to the extent of the injury done to the spinal marrow, and not to the operation, which, we think, offers the best, or rather the only chance of success. Whether the operation be ventured on or not, cupping, leeching, blisters, and the proper antiphlogistic measures, will be indispensible till the fever subside, and then a drain should be established near the part, to obviate the effects of local inflammation.

We have now presented our readers with an analysis of all except that part of the work which was formerly reviewed by us—namely, the section on Compound Dislocations of the Ancle-joint. It has been obvious, that it was for the interest of the reader that we should confine ourselves strictly to analysis, without squandering our own, or our readers' time in useless criticism. Such a mass of important practical matter was never, we believe, before laid open to the public; and Sir Astley Cooper's work will continue to exercise that influence on the surgical profession at large, when the author has mouldered in the dust, which the living author has so long exercised (and which we hope he will still long exercise) within the sphere of his personal acquaintance and practice.

